# Upregulation of miR-142 in papillary thyroid carcinoma tissues: a report based on in silico and in vitro analysis

**DOI:** 10.22099/mbrc.2022.43947.1757

**Published:** 2022

**Authors:** Sepehr Valizadeh, Mojtaba Zehtabi, Neda Feiziordaklou, Zahra Akbarpour, Amir Mahdi Khamaneh, Mortaza Raeisi

**Affiliations:** 1 Department of Internal Medicine, School of Medicine, Tabriz University of Medical Sciences, Tabriz, Iran; 2 Hematology and Oncology Research Center, Tabriz University of Medical; 3 Sciences, Tabriz, Iran; 4 Rahat Breathe and Sleep Research Center, Tabriz University of Medical Sciences, Tabriz, Iran; 5 Department of Molecular Medicine, Faculty of Advanced Medical Sciences, Tabriz University of Medical Sciences, Tabriz, Iran

**Keywords:** Papillary thyroid cancer, MicroRNA, Bioinformatics, Microarray data analysis

## Abstract

Papillary thyroid carcinoma (PTC) accounts for approximately 80% of all human thyroid malignancies. Recently, there has been a dramatic rise in the prevalence of thyroid cancer all over the globe. Through analysis of the GEO database, GSE104005, the authors of the current research were able to determine the differential expression of microRNAs (DEMs) as well as their target genes. Real-time PCR was used on a total of 40 samples, 40 of which were from PTC samples and 40 from normal tissues, in order to validate the discovered DEMs and the genes. Gene Ontology (GO) categories were identified, and KEGG was used to conduct pathway enrichment analysis. The multiMiR R package was used to predict target genes of DEMs. Mir-142 was found to be overexpressed in PTC samples, as compared to normal tissues, and this was validated by the identification and validation. In addition, metal ion binding and the cellular response to metal ions were identified as essential pathways in the carcinogenesis of PTC. This demonstrates their significance in the development of this malignancy. The results of our research will serve as the foundation for further research in the area of miRNA-based cancer treatment.

## INTRODUCTION

It has been proven that microRNAs may have function in the development and progression of papillary thyroid carcinoma (PTC), a type of cancer which is rising all over the world [1]. According to the reports from the American National Cancer Institute, the median age of patients when they were first diagnosed with thyroid cancer was 50 years old during the years 2005-2009 [2]. In 2012, it was estimated that there were 56,460 new cases of thyroid cancer and 1,780 deaths caused by the disease in the United States [3]. 

The majority of PTCs may be successfully treated by surgical excision, which is then followed by adjuvant radioactive iodine (RAI) therapy [4]. As a result, the 5-year survival rate is more than 95 % [5]. However, a portion of patients do not react to RAI treatment and/or proceed to metastatic malignancy; in these instances, the prognosis is poor, and the percentage of patients who survive for 10 years or more is reduced to 10% [6]. Despite the fact that these individuals have been subjected to a number of different therapies, very minimal gains have been realized, and there are currently no efficient treatments. The use of differential miRNA expression in PTC vs normal tissue samples as diagnostic and prognostic markers of thyroid cancer has been described before. The mechanism of action for miR-150, miR-422a, miR-18a, and miR-19a in human thyroid cancer has been studied to some extent; however, there are still a number of miRNAs that may be biologically relevant for the development of thyroid cancer [7]. MicroRNAs are small non-coding RNAs that are less than 21 nucleotides in length. They are responsible for regulating gene expression at the post-transcriptional level and are involved in a wide variety of biological processes, including the development of tumors [8]. By controlling the expression of its target oncogene(s) and tumor suppressor(s), a particular miRNA may perform either the role of an oncogene or a tumor suppressor [9]. In most cases, when miRNAs attach to the 3'-untranslated regions (3' UTRs) of the mRNAs that they are targeting, this results in the destruction of the mRNA or the inhibition of its translation [10]. 

The current research focused on investigating the dysregulation of miRNAs in PTC. We carried out miRNA and gene microarray analysis on PTCs, and then we confirmed the results of that research through real-time PCR method.  In addition, we conducted a functional analysis of the target genes that we had obtained in order to identify potential pathways that implicated in the progression of PTC.

## MATERIALS AND METHODS

**Data collection and preprocessing: **The microRNA expression profile known as GSE104005 was obtained by accessing the Gene Expression Omnibus (GEO) database at the following address: https://www.ncbi.nlm.nih.gov/geo/ [11]. A quantile normalization was carried out so that the dataset could be normalized [12]. This dataset was constructed using the Illumina HumanHT-12 WG-DASL V4.0 R2 expression beadchip. It consisted of 30 samples of thyroid cancer and 6 samples of non-neoplastic thyroids (https://www.ncbi.nlm.nih.gov/geo/ query/acc.cgi?acc=gse104005). In this particular dataset, we used 20 PTC samples and compare the miRNA expression patterns to those of control samples.

**Identification of DE-miRNAs and DEGs: **R software, version 4.1.1, was used to analyze the data. In this regard, the data were normalized by the use of the quantile normalization technique. Box plots was also generated using normalized data with the assist of Gplot program (Fig. S1). Downloading and using the Limma (version 3.483), data.table (version 1.14), and GEOquery (version 2.60) allowed us to identify of DE-miRNAs as well as DEGs. As a cutoff criterion in our study, the values consisted of a p.value less than 0.05 and a |Log2FC| value of more than 2, were considered statistically significant. 

**Identification of target genes: **Once we obtained a list of potential microRNAs, the next step was to identify the genes that were targeted by these microRNAs. This will allow us to do further analysis depending on the results of those investigations, such as gene ontology. In order to anticipate target genes, the Multimir package [13] was used, which is a software that predicts target genes from the mirTarbase and Tarbase databases. The processes that were carried out resulted in the discovery of a significant number of genes that may be put to use in the investigation of cellular and molecular mechanisms involved in the tumorigenesis of PTC.

**Functional Enrichment Analysis of Putative Target Genes: **Identified target genes were put into the Funrich 3.1.3 program as a supplementary software to the Gene Ontology (GO) and KEGG pathway analyses. A significance level of p-value < 0.05 was utilized as the inclusion criteria for both GO and KEGG enrichment analysis. Kyoto Encyclopedia of Genes and Genomes, or KEGG, is an integrated database resource for the biological interpretation of genome sequences and other high throughput data [14].

**PPI network construction: **After performing functional enrichment analysis, a network of protein-protein interactions was constructed on the basis of the software packages STRING and Cytoscape [15, 16]. The target gene was selected from the STRING database with a score of more than 0.9 (maximum confidence), and the interaction network was visualized with the help of the program Cytoscape. In addition, the CytoHubba package was used to locate genes that function as hubs. The PPI network contains selected genes known as hub genes. Each hub gene has a large number of interaction partners and thus acts as a critical node in the network of gene interactions. CytoHubba was able to identify hub genes by overlapping the top 10 genes, which was one of the 11 different classification strategies.

**Sample collection: **Patients diagnosed with PTC who were scheduled to undergo thyroidectomy at the Imam Reza hospital provided a total of 40 thyroid tumor tissue samples for collection. In addition, 40 samples of non-neoplastic thyroid tissues were retrieved from the same hospital. It is important to note that both the PTC samples and the normal samples were kept frozen in liquid nitrogen before being used. Pathologists also confirmed the histopathological classifications of the tumors.

**RNA extraction and cDNA synthesis: **Using the Trizol reagent (Ambion life technologies, UK), total RNA was isolated from the 300 mg pure human PTC specimens. The extraction was carried out in accordance with the procedure provided by the manufacturer. The Nanodrop tool (Spectrophotometer 2000/2000c) was used to determine the amount of purified RNA measured in ng/ul as well as any potential protein or mineral residue contaminations. cDNA was synthesized by following the temperature protocol provided by the manufacturer of the AnaCell cDNA synthesis kit (Lot No: cs0025), which was as follows: 70°C for 5 minutes, 37°C for 60 minutes, and then 70°C for 5 minutes. The stem-loop method that was offered by the Biomir high sensitivity microRNA kit (Zistroyesh. Co/Iran) was used in the complementary DNA synthesis process for each target as well as the housekeeping miRNA (U6).

**Reverse transcription-quantitative Q-PCR: **PCR was carried out with the use of a SYBER Green PCR kit (manufactured by AnaCell in Iran) and miR-specific primers, as well as Rad17 and Rrm2b primer. Primers for stem-loop RT amplification of miRNAs were included in the kit (manufactured by Zistroyesh. Co. in Iran) and used in accordance with the instructions supplied by the manufacturer. The following describes the conditions for the PCR: 15 minutes of pre-denaturation at 95°C., followed by 40 cycles of denaturation at 95°C. for 20 seconds, an annealing-extension step at 60°C. for 60 for 60 seconds, and a final melt analysis step at 55 degrees Celsius to 95°C. 

The *GAPDH* (glyceraldehyde-3-phosphate dehydrogenase) gene and the U6 gene, respectively, were used to normalize the Rad17 and Rrm2b as well as the miRNAs. These genes were considered to be housekeeping genes. Every RT-qPCR test was run in triplicate to ensure accuracy. Table 1 has a detailed listing of the primers for Rad17 and Rrm2b, as well as GAPDH.

**Table 1:** List of primers related to genes and housekeeping gene

**Name**	**Sequence**	**Length**	**TM**	**GC**	**Position**	**Product length**
F (RAD17)	TCCAAACTCAGCTATTGCCAT	21bp	57	43	2174, Exon 16	118bp
R (RAD17)	AATCTTCCAAAGTGTCGCTTC	21bp	57	43	2271, Exon 17	
F (RRM2B)	ATGGCTTTCAAATTCTCATCG	21bp	55	38	596, exon 4	166bp
R (RRM2B)	CTATCTGCTATCCATCGCAAG	21bp	56	47	741, exon 5	
F (GAPDH)	TCTGACTTCAACAGCGACACC	21bp	60-64	-	Exon 7	117bp
R (GAPDH)	GTTGCTGTAGCCAAATTCGTT	21bp	57-60	-	Exon8	

**Statistical Analysis: **The ΔΔCt method was used for the investigation of gene expression. The formula for calculating ΔCt is as follows: ΔΔCt=ΔCt (Ct target gene–ΔCt ref. housekeeping gene; treated sample) - (Ct target gene –Ct ref. gene; untreated control). When comparing the expression levels of the target gene to those of the reference gene, the Ct values were used, and the fold changes reflected the relative quantification. The Shapiro-Wilk test was used to determine whether or not the given data were normally distributed. A paired t-test was carried out since the data distribution for Rad17 and Rrm2b, as well as miR-142 and miR-420, were all normal. A p-value of less than 0.05 was regarded as statistically significant. Statistical analyses and the generation of boxplots were carried out with the help of the GraphPad Prism program, version 8.0.2. Using Pearson's r, we determined the significance of the correlations between the fold changes (FCs) of Rad17 and Rrm2b as well as the levels of miRNA.

## RESULTS

**Figure 1:**
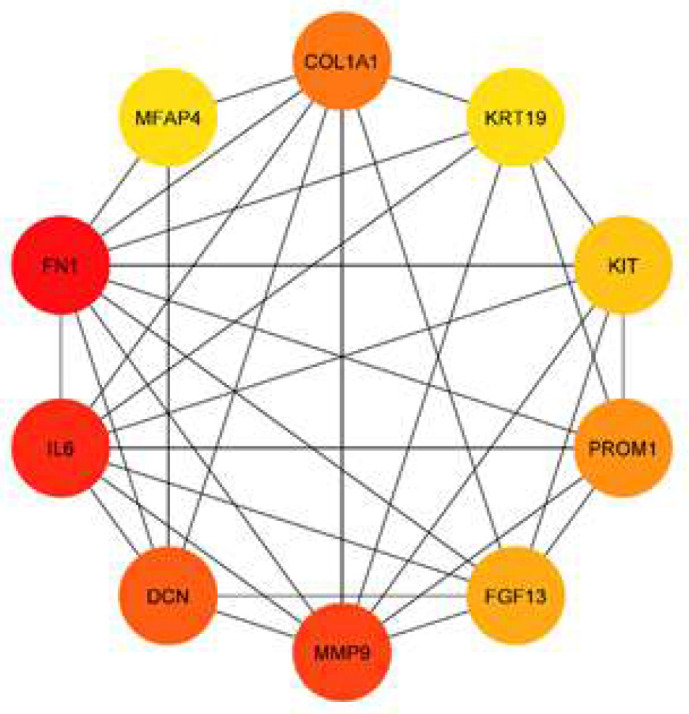
Constructed protein-protein interaction and selected hub-nodes

There were a total of three miRNAs that were found to have differential expression, and they were all elevated by a LogFC of more than 2 (miR-142, miR-421, and miR-221). During this time, a total of 64 genes with differential expression were discovered, 19 of which were upregulated and 45 of which were downregulated (with a |Log FC value|>than 3) (Table 2). Moreover these data were also normalized which is shown in Figure 1 and normalized data were used to further bioinformatics steps. When the findings of DEGs and anticipated target genes were compared, it was discovered that RRM2B and RAD17 were the genes that were targeted by miR-142 and miR-421, respectively. As a direct consequence of this, the previously described microRNAs and genes have been chosen for investigation in PTC tissue samples.

An enrichment analysis using GO functional and KEGG pathway analysis were carried out on the genes that were just highlighted as being potential targets. The following table outlines the enriched GO functions that are associated with the target genes: metal ion binding (GO:0046872) in the (MF) category, cellular response to metal ion (GO:0071248) in the (BP) category, and collagen-containing extracellular matrix (GO:0062023) in the (CC) category. In addition, the enriched KEGG pathways for target genes of dysregulated miRNAs found 5 signaling pathways by adj.p.value< 0.05. These signaling pathways include Retinol metabolism, Tyrosine metabolism, the Metabolism of xenobiotics via cytochrome P450 route, and the drug metabolism pathway. In addition to evaluating the functions of protein products that were created from the genes that were evaluated, the interaction of these products was explored. The results of this investigation showed that there is a vast network of molecular links between proteins.

According to the findings of the STRING database, a number of the target genes interacted with one another. In order to maximize exposure, only the 10 hub nodes that received the best possible scores were chosen (Fig. 1). The following genes have been recognized as hub genes: FN1, IL6, MMP9, DCN, COL1A1, PROM1, FGF13, KIT, KRT19, and MFAP4 (Table 3). Out of all of these genes, FN1 had the highest node score (1328).

**Figure 2:**
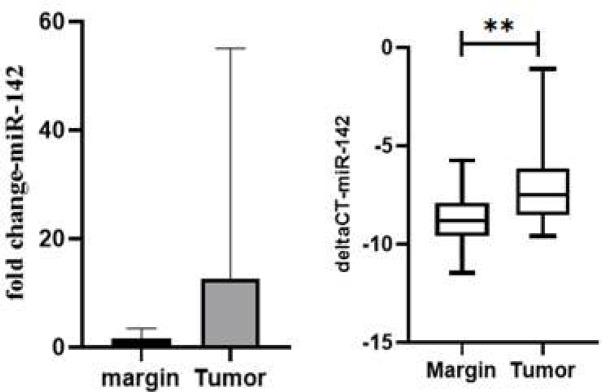
Relative expression levels of miR-142 in PTC vs. control thyroid tissues

In contrast to the normal tissues that were adjacent to the PTC, the expression level of miR-142 was found to be 2.76 times higher in the PTC tissues. This result, which was statistically significant with a p-value of 0.049, is shown in figure 2.

**Table 2:** Differentially expressed genes identified between PTC and normal adjacent tissues

**Gene name**	**Log FC**	**p.value**	**Adj.p.value**	**Gene name**	**Log FC**	**p.value**	**Adj.p.value**
ZCCHC12	4.508553	0.001644	0.044631	OCA2	-3.22103	1.01E-06	0.000328
SERPINA1	3.839261	0.001304	0.03987	APOD	-3.2445	4.16E-05	0.004368
CLDN16	3.552258	0.000588	0.024969	KCNIP4	-3.24741	4.94E-06	0.000967
LAMB3	3.535066	0.001415	0.041006	GDF10	-3.29988	6.45E-08	5.26E-05
ARHGAP36	3.495579	0.014246	0.149301	AOX1	-3.3166	5.01E-07	0.00021
DCSTAMP	3.447977	0.014667	0.151613	DPT	-3.3601	7.56E-05	0.006644
MIR221	3.394567	0.000235	0.013759	EDN3	-3.39653	0.000787	0.029563
HMGA2	3.389395	9.41E-06	0.001589	SEMA3D	-3.39872	0.000731	0.028226
FN1	3.352628	9.35E-06	0.001589	PLA2R1	-3.40243	8.06E-08	5.92E-05
GABRB2	3.325896	0.00058	0.024749	TFF3	-3.41232	0.000943	0.032978
HLA-DQB2	3.191091	0.005331	0.08893	ELAPOR1	-3.4718	3.12E-07	0.000151
CITED1	3.182578	0.001858	0.048169	SRARP	-3.48399	3.55E-12	3.48E-08
MUC21	3.163062	0.008502	0.113571	AGR3	-3.48904	6.08E-08	5.11E-05
LIPH	3.156156	0.00043	0.020113	GPC3	-3.49104	2.69E-08	2.92E-05
FN1	3.150229	3.81E-06	0.000788	DPP6	-3.50692	0.000653	0.026401
METTL7B	3.133466	1.48E-07	8.88E-05	OGN	-3.50747	1.05E-06	0.000328
NGEF	3.093314	0.00188	0.048405	LIX1	-3.52098	9.68E-06	0.001598
TENM1	3.039968	0.002742	0.061156	PKHD1L1	-3.52818	0.000267	0.014787
PDZK1IP1	3.030494	0.006582	0.099435	KCNA1	-3.63596	8.70E-11	3.20E-07
KCNIP4	-3.00341	1.05E-05	0.001663	MT1H	-3.66233	0.000484	0.021988
AGR3	-3.00742	6.29E-10	1.42E-06	ODAM	-3.66702	4.02E-05	0.004279
VIT	-3.00901	1.85E-06	0.000485	RELN	-3.6861	3.18E-05	0.00371
OGN	-3.01036	2.50E-06	0.000596	PI16	-3.7009	2.89E-06	0.000654
TCEAL2	-3.02632	0.001951	0.049462	CDH16	-3.80527	0.000192	0.012179
CHRDL1	-3.0385	5.01E-06	0.000974	ZNF804B	-3.83449	0.000205	0.012513
VIT	-3.05628	1.36E-06	0.000388	LRP1B	-3.95376	1.68E-06	0.000452
PGA5	-3.10873	2.96E-06	0.000659	MT1H	-3.97437	0.000402	0.019347
LYVE1	-3.14714	3.33E-10	8.16E-07	CRABP1	-4.02482	5.01E-05	0.00501
PLA2R1	-3.18258	1.07E-06	0.000332	MYOC	-4.04499	6.29E-12	4.62E-08
RERGL	-3.18576	6.74E-05	0.006109	ADH1B	-4.07759	1.07E-09	2.26E-06
CA4	-3.20718	0.001955	0.049512	CCL21	-4.2797	0.000141	0.009811
LYVE1	-3.21914	3.32E-12	3.48E-08	SCARA5	-4.7367	2.75E-07	0.000137

**Table 3:** List of hub nodes and their scores

**Rank**	**Name**	**Score**
1	FN1	1318
2	IL6	1063
3	MMP9	864
4	DCN	567
5	COL1A1	554
6	PROM1	417
7	FGF13	398
8	KIT	330
9	KRT19	314
10	MFAP4	314

**Figure 3:**
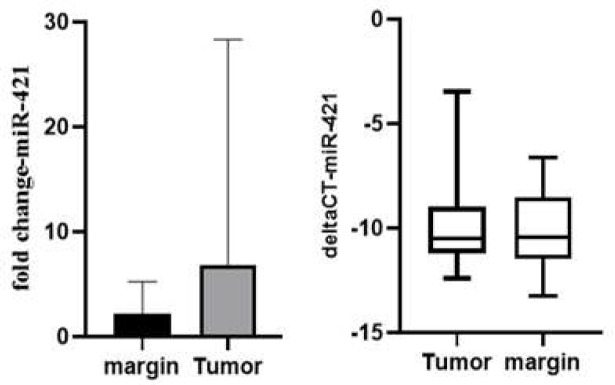
Relative expression levels of miR-421 in PTC vs. normal thyroid samples

A 1.22-fold increase in the expression level of this miRNA was discovered in the PTC in comparison with the non-cancerous adjacent samples, as can be seen in figure 3. This was determined by evaluating the levels of miR-421. However, owing to the fact that the p-value was 0.777, the rise in the expression was not considered to be statistically significant.

**Figure 4:**
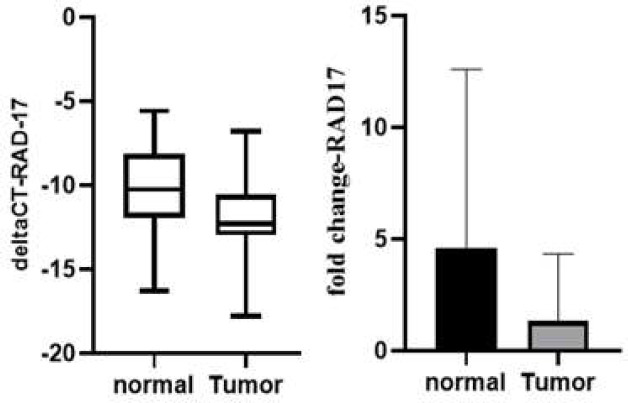
Relative expression levels of RAD17 in PTC vs. control thyroid samples

In the analysis of RAD17 mRNA levels in the PTC and adjacent normal tissues, it was shown that the expression levels in the PTC samples were 3.108 times lower than those in the surrounding normal tissues. This result, which did not reach the level of statistical significance (p=0.151), is shown in figure 4.

**Figure 5:**
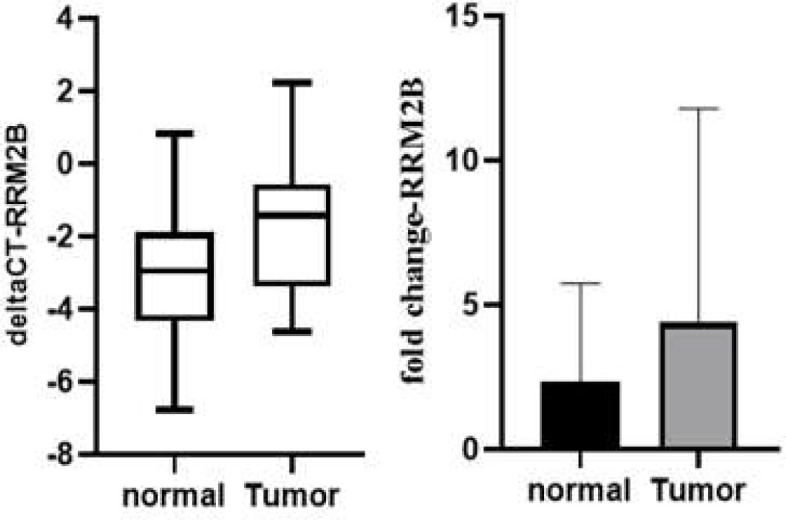
Relative expression levels of RRM2B in PTC vs. normal thyroid tissues

In the instance of RRM2B, an investigation of the expression fold-change of this gene in PTC samples revealed a 2.084-fold overexpression in comparison to normal sample levels. This increase, on the other hand, did not meet the criteria for statistical significance, since its p-value was just 0.101 (Fig. 5).

## DISCUSSION

MicroRNAs are involved in a wide variety of biological and metabolic processes, including the differentiation, proliferation, survival, and development of cancerous cells [17]. They are endogenous single-stranded non-coding RNAs that are less than 21 nucleotides in length, and they inhibit the production of genes by preferentially attaching to the complementary 3' UTR of the mRNAs that they target [18]. This is accomplished by incorrect base-pairing. 

We compared the gene expression profile with the miRNA profile in order to improve the accuracy of the predicted target genes. Then, we restricted the target genes to those known to be shared by both the differentially expressed genes (DEGs) and the anticipated target genes. This helped us achieve our goal. In the current work, we investigated a dataset named GSE104005 from the Gene Omnibus (GEO) database. This dataset included 30 PTC samples and 6 normal adjacent tissues. This dataset was compromised of both non-coding and gene expression profiling platforms.  According to the findings of this microarray dataset analysis, a total of 64 genes and 3 miRNAs were found to be in a deregulated state. PPI construction, hub node identification for PPI, and functional analysis of the in common target genes and discovered DEGs were all carried out. In the end, the microRNAs mir-142 and miR-421, as well as the genes RAD17 and RRM2B that are likely to be their targets, were chosen for further verification using real-time PCR. According to the results of an in-silico investigation, the levels of miR-142 and miR-421 had increased by a LogFC of 2.65 and 2.51, respectively. The RAD17 and RRM2B mutations had values of -2.61 and -2.71, respectively, for these changes. On the other hand, the results of our real-time PCR experiments indicated that the levels of the miRNAs miR-142 and miR-421 had increased, corroborating the findings of our bioinformatics studies. However, the change in expression of miR-142 was the only one that could be considered statistically significant. In contrast to the results of our bioinformatic study, the expression level of RRM2B was found to have increased when examined at the level of the mRNA. However, the significance rate did not meet the statistical requirements required for acceptance. In the end, RAD17 was down-regulated, which was consistent with the results of our in silico research; nevertheless, the finding did not prove to be statistically significant in our investigation. This is the first research that we are aware of that demonstrates that miR-142 is elevated in PTC, to the best of our knowledge. 

Studies have shown, for the most part, that miR-142 has a paradoxical expression profile pattern in a variety of cancers. Downregulation of miR-142–3p was shown to have a role in the development of thyroid tumors by Colamaio et al [19]. Mir-142 did this by controlling the expression of their target genes, which included ASH1L and MLL1 [19]. Furthermore, Mansoori et al. discovered that the expression of miR-142-3p is downregulated in ER-positive breast cancers, and that restoring its expression in ER-positive breast cancer cells may reduce and induce cell viability and apoptosis, respectively, in the cells [20].  However, Wang et al. demonstrated that miR-142-5p acts as a growth inhibitory miRNA and has a significant role in preventing the development of non-small cell lung cancer by targeting PIK3CA [21]. This was accomplished by miR-142-5p's ability to target PIK3CA. When it comes to functional analysis, our findings demonstrated that the majority of genes play important roles in the pathways that are associated with metal ions. It is important to note that Gerwen et al. demonstrated that increased manganese levels are found in thyroid cancer tissues, which demonstrates the fact that urgent need is required for future studies. This is especially true when considering the increasing exposure of the general population to a variety of environmental pollutants, including metal ions, and the prevalence of thyroid cancer around the world [21] .

In conclusion, a number of microRNAs that are associated with thyroid cancer and the genes that they target were found, and the validity of mir-142 was established using the real-time PCR technique. We can further understand the involvement of microRNAs in papillary thyroid carcinogenesis by basing our analysis on the activities of the target genes, and we can also evaluate whether or not microRNAs have the potential to be used as biomarkers for early diagnosis. Further research is required to further confirm the accuracy and specificity of our data since our study only used a small number of samples and our findings did not see a p-value that was statistically significant. In addition, our results provide a solid foundation upon which to build future investigations in the area of miRNA-based cancer treatment. In the end, since the preceding research provided evidence to support the premise of the current study by demonstrating the significance of the metal ion binding pathway in the carcinogenesis of PTC, it is imperative that this pathway be further investigated in further studies.

## Conflict of Interest:

 The authors of this research have indicated that they have nothing to report about potential conflicts of interest in relation to this publication.
